# A Neural Model for Temporal Order Judgments and Their Active Recalibration: A Common Mechanism for Space and Time?

**DOI:** 10.3389/fpsyg.2012.00470

**Published:** 2012-11-02

**Authors:** Mingbo Cai, Chess Stetson, David M. Eagleman

**Affiliations:** ^1^Department of Neuroscience, Baylor College of MedicineHouston, TX, USA; ^2^Department of Neuroscience, California Institute of TechnologyPasadena, CA, USA; ^3^Department of Psychiatry, Baylor College of MedicineHouston, TX, USA

**Keywords:** opponent processing, synaptic scaling, temporal order judgment, recalibration, motion aftereffect

## Abstract

When observers experience a constant delay between their motor actions and sensory feedback, their perception of the temporal order between actions and sensations adapt (Stetson et al., 2006). We present here a novel neural model that can explain temporal order judgments (TOJs) and their recalibration. Our model employs three ubiquitous features of neural systems: (1) information pooling, (2) opponent processing, and (3) synaptic scaling. Specifically, the model proposes that different populations of neurons encode different delays between motor-sensory events, the outputs of these populations feed into rivaling neural populations (encoding “before” and “after”), and the activity difference between these populations determines the perceptual judgment. As a consequence of synaptic scaling of input weights, motor acts which are consistently followed by delayed sensory feedback will cause the network to recalibrate its point of subjective simultaneity. The structure of our model raises the possibility that recalibration of TOJs is a temporal analog to the motion aftereffect (MAE). In other words, identical neural mechanisms may be used to make perceptual determinations about both space and time. Our model captures behavioral recalibration results for different numbers of adapting trials and different adapting delays. In line with predictions of the model, we additionally demonstrate that temporal recalibration can last through time, in analogy to storage of the MAE.

## Introduction

**Figure 1:**
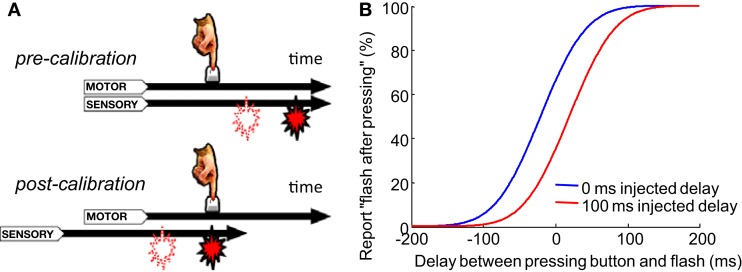
**Recalibration of temporal order judgment after adapting to a constant delay between motion and sensory event**. **(A)** A flash consistently appears with a fixed delay (*filled icon*) after a button press. If a flash is suddenly presented with a shorter delay (*open icon*), it will be perceived as appearing before the press after calibration. Stetson et al. (2006) proposed that this is due to the relative shift of the motor and sensory time lines (i.e., temporal expectations; reproduced from Stetson et al., 2006, with permission; **B)**. A cartoon of psychometric curves of a participant judging the temporal order between a button press and a flash. In the control condition, before each test trial, participants are presented with a flash immediately after each of 3–5 presses of the button. Then, in a testing trial, they report the perceived temporal order between their press and a flash that is randomly presented before or after the press. Data from the testing trials are plotted in blue. The red curve represents the adaptation condition, in which a constant 100 ms delay is injected between the flash and each of the 3–5 presses before the test trials. Curves based on average data from participants in Stetson et al. (2006).

Animals must correctly judge the order of self-generated motor acts (output) and sensory events (input). This judgment is necessary for attributing the source of the sensory event to the animal’s own action, or instead to another agent (e.g., a predator). Correctly making these temporal order judgments (TOJs) can be challenging because the processing speeds in sensory pathways can fluctuate, both at short time scales (e.g., from changes in lighting; (Purpura et al., 1990)) and at long time scales (e.g., from a lengthening of motor and sensory signal transmission times due to body growth). To come to the correct conclusion about the order of events, the brain must have a mechanism to recalibrate its expectation of the normal processing latencies associated with action and sensation (Stetson et al., 2006; Eagleman, 2008). Previous research has shown that the perceived interval between a voluntary action and subsequent sensory effect can contract after exposure to a constant delay of the effect (Cunningham et al., 2001; Eagleman and Holcombe, 2002; Haggard et al., 2002). This contraction was proposed to result from an “intentional binding” between the action and the resulting sensation, drawing the perception of the timing of the two events closer together (Haggard et al., 2002). Stetson et al. (2006) proposed that these results could be alternatively caused by a shift of the perceived time of sensory events relative to the perceived time of actions (Figure 1A) – in other words, by a recalibration of the expected timing relationships. By repeatedly injecting a constant delay between a participant’s button press and a consequent flash, TOJs of the button press and flash could become reversed: after adapting to delayed flashes, participants would judge a flash as having come *before* their press, even in a time range in which the flash physically followed the press (Stetson et al., 2006, Figure 1B). Similarly, other studies demonstrate that a constant delay between a pair of cross-modal events (such as a beep and a flash) can alter the perceived simultaneity between the two modalities (Fujisaki et al., 2004; Vroomen et al., 2004; Hanson et al., 2008). Although the recalibration of timing judgments has been clearly demonstrated behaviorally, researchers have only started to propose models for the underlying neuronal mechanism recently (Roach et al., 2011). Here we propose a neural model to explain TOJs and their recalibration. Further, we test predictions of the model on the number of adapting trials and on the “storage” of temporal recalibration. With these data in hand, we will demonstrate that the same model can be modified to explain recalibration in the spatial domain – that is, the motion aftereffect (MAE).

## Materials and Methods

### A model for temporal order judgments and their recalibration

To capture the psychophysical data of temporal recalibration (Stetson et al., 2006), we will draw upon three ubiquitous neural motifs: information pooling, opponent processing, and synaptic scaling.

We will begin by focusing on information pooling and opponent processing. Generally, comparing the weighted sums of the responses of low-level stimulus-tuned neurons seems to be a general mechanism for higher level neural populations to achieve a perceptual judgment (Shadlen et al., 1996). With this in mind, we will explore whether TOJs can be explained by a model which capitalizes on neural pooling and opponent processing. Details of the model are described in the next section, but the essence is simply described: (1) a population of delay-tuned neurons encodes different delays between motor actions and sensory inputs, and (2) the outputs of these delay-tuned neurons are pooled into two higher level populations that compete with each other to reach a decision. In this way, the higher level populations pool evidence of “motor act *before* sensory input” or “motor act *after* sensory input.” These jointly account for the two alternatives in a TOJ, forming the basis of causality judgments (Eagleman and Holcombe, 2002).

If correct, this model for TOJs suggests that the findings in Stetson et al. (2006) and other studies involving adaptation to asynchrony between cross-modality sensory events (Fujisaki et al., 2004; Vroomen et al., 2004; Hanson et al., 2008) may reflect a temporal analog to the MAE, potentially offering a unified explanation for both. We hypothesize that identical neural mechanisms may underlie judgments of both time and space, leading to analogous illusions in both domains. To capture the dynamic recalibration of such judgments, our model incorporates synaptic scaling. That is, if there is too little activity impinging on a neuron, the neuron globally scales up the synaptic strengths of its inputs; similarly, in response to too much activity, it globally scales down the input weights (Turrigiano, 1999; Turrigiano and Nelson, 2000; Ibata et al., 2008). We show that the synaptic scaling of the neurons that pool information from lower level delay-tuned neurons provides a way to recalibrate the set-point for the system, such that the perceptual decision adapts according to recent experience.

#### Temporal order judgment

**Figure 2:**
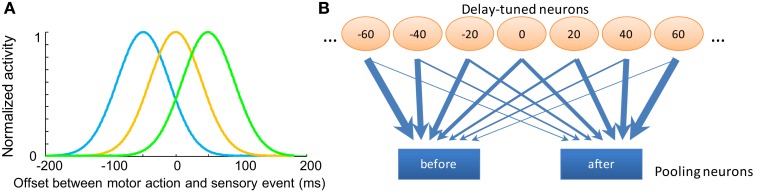
**Opponent processing model for temporal order judgments**. **(A)** The tuning curves of several hypothetical “delay sensitive” neurons which have the strongest responses for specific delays between motor and sensory events. **(B)** Diagram of the pooling opponent processing circuits. Orange circles represent the hypothetic lower level delay sensitive neurons with different preferred delays between motor action and sensory input. Blue rectangles represent pooling modules with different input weight patterns from the lower level neurons; they selectively receive stronger synapses from neurons coding for “before” or “after,” respectively. The width of the arrows indicates the strength of the weights. These pooling modules compete with each other to reach a decision; the one with stronger activity will represent the final judgment of temporal order.

Neurophysiologic studies demonstrate multisensory integration neurons in cat superior colliculus: these neurons fire maximally at particular stimulus onset asynchrony (SOA) between bi-modal sensory inputs, such as audiovisual stimuli (Meredith et al., 1987). As the SOA deviates from the “optimal” interval, the spiking rates decrease monotonically to the level triggered by single-modality input, forming a bell-shaped tuning curve with respect to the SOA. Although we are not aware of literature on the same pattern of neural tuning for delays between the motor efference copy and the sensory input in mammals, neurons with complex encoding patterns for different delays have recently been found in dorsolateral prefrontal cortex (DLPFC) and caudate nucleus (CN) during a visuomotor task (Jin et al., 2009). Here, we postulate the existence of low-level neurons sensitive to different delays between motor action and sensory input (a negative delay means sensory input precedes motor action). Different populations of neurons give maximum responses to different delays between motor and sensory events (Figure 2A), an analog to the visual motion direction tuned neurons in the MT region of primates (Albright, 1984). We denote the spiking rates of *n* delay sensitive neurons in response to an interval between consecutive action and sensory events as a vector $\vec{X}=\left[x_{1},\;x_{2},\;x_{3},\;...,\;x_{n}\right]$, where x_i_ is the firing rate of the *i*^th^ neuron. The tuning curve of each neuron is modeled by a Gaussian function with its standard deviation as a free parameter.

$$x_{i}=F\exp\left[-{\left(t-\tau_{i}\right)}^{2}∕2\sigma^{2}\right]+\epsilon$$

In the above equation, *F* is the maximal firing rate, *t* is the real delay between action and sensory event. τ_i_ is the preferred delay of the *i*^th^ neuron. σ is the width of tuning curve common for all neurons. ε is additive noise approximating Poisson noise (Fano factor of 1).

These populations then feed into two pooling modules with different synaptic weights (Figure 2B). The two pooling modules selectively receive inputs from the low-level neurons that are most informative for “before” or “after” order judgments. The synaptic weight of the *i*^th^ low-level neuron to the high-level pooling neurons encoding for “after” is determined by a cumulative Gaussian distribution:

$$W_{Ai}=\Phi \left(\tau_{i};0,\lambda^{2}\right)=\frac{1}{\sqrt{2\pi \lambda^{2}}}\overset{\tau_{i}}{\underset{-\infty }{\int }}e^{-v^{2}∕2\lambda^{2}}dv$$

where *τ_i_* is again the sensitive delay of the *i*^th^ low-level neuron. λ is a free parameter describing the standard deviation of the cumulative Gaussian function. And the synaptic weight to the high-level pooling neurons encoding for “before” is determined by the reverse of such a cumulative Gaussian distribution:

$$W_{Bi}=\Phi \left(-\tau_{i};0,\lambda^{2}\right)=1-\frac{1}{\sqrt{2\pi \lambda^{2}}}\overset{\tau_{i}}{\underset{-\infty }{\int }}e^{-v^{2}∕2\lambda^{2}}dv$$

**Figure 3:**
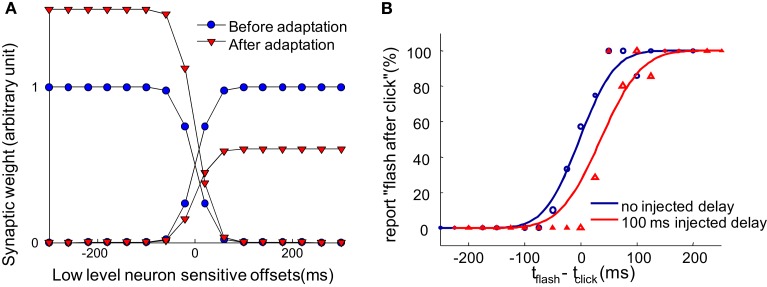
**Synaptic scaling at the single neuron level in the pooling populations gives rise to recalibration**. **(A)** Illustration of the change to the weights of the pooling modules (the one-directional arrows pointing to the blue rectangles in Figure 2B) after the system is constantly exposed to a 100 ms delay between an action and a sensory event. Before adaptation, the weights of the pooling modules are balanced, as shown by the blue circles. After adapting to a constant positive delay, the pooling populations encoding for “flash after press” globally decreases their weights and the pooling populations encoding for “flash before press” globally increase their weight, as shown by the red triangles. **(B)** The psychometric curve obtained by simulating the network proposed in Figure 2B. The psychometric curve shifts so that a delayed sensory event is perceived simultaneously with the motor action. The finite slope of the curve comes from the Poisson-like noise on the lower level neurons and the gradual scaling of synaptic weights after a constant injected delay.

The lines with blue circles in Figure 3A provide a cartoon description of the pooling synaptic weight pattern. The exact form of this synaptic weight pattern is not critical. The essence is that the system looks for the difference in activity between populations encoding for “before” or “after” (Figure 2B) to obtain a decision. This sort of decision-making mechanism is supported by single-neuron recording studies in monkeys performing sensory discriminations tasks (Newsome et al., 1989; Schall, 2001). Shadlen et al. (1996) proposed that perceptual decisions are made by integrating the difference in spiking rates from pools of low-level neurons selectively tuned to different motion directions. For example, in a motion direction judgment task, in which monkeys must decide whether more dots move up or down in a group of randomly moving dots, the difference between the weighted sum of the firing rates of low-level neurons sensitive to upward motion and those sensitive to downward motion can serve as the basis of the decisions (Shadlen et al., 1996; Gold and Shadlen, 2001). fMRI experiments support that a similar mechanism is implemented in humans (Heekeren et al., 2004). These findings suggest that a comparison of the outputs of different pools of selectively tuned lower level sensory neurons is a general mechanism for computing perceptual decisions.

The two pooling modules take the weighted sum of the firing rates from the delay-tuned neurons as input. The firing rate is a sigmoid transformation of the input:

$$\begin{array}{llll} & f_{\text{q}}=S\left(\overset{n}{\underset{i=1}{\sum }}w_{qi}\cdot x_{i}\right),\left(q=\text{A},\text{B}\right) & & \text{(1)}\\ & S\left(y\right)=\frac{2\cdot m}{1+{e^{-}}^{\frac{y-m}{m}}} & & \end{array}$$

where *m* is the average input to the pooling modules for a broad range of delays between action and sensory events.

After pooling the evidence of the delay-tuned neurons’ weighted activities, the decision of the model is simply determined by the pooling module with the higher activity. Note that although we use sigmoid function to model the non-linear transfer function of the pooling neurons, the detailed shape of this function is not critical to the relative relation between *f*_A_ and *f*_B_, and therefore the decision of the system.

The range of the optimal delays of the low-level neurons is bounded. In reality, the temporal offset between two events can exceed the range of the delay-tunings. We take this bounded encoding range of the low-level neurons to be a reasonable assumption for several reasons. First, the multisensory neurons found in cat superior colliculus also show a limited range of preferred SOAs (Meredith et al., 1987). Second, in audiovisual integration studies, the McGurk effect is only found when the temporal disparity between audio and visual stimuli vary in a range of hundreds of milliseconds and disappears with larger temporal disparity (van Wassenhove et al., 2007), indicating a limited time range of neural processing for attributing a common source for multisensory stimuli. Finally, a limited range of SOAs is suggested by environmental constraints, because events in two modalities that belong to the same cause are more likely to arrive close in time. A similar situation applies to motor-sensory events: an effect related with an action is likely to arrive within a limited time range of the efference copy (Eagleman and Holcombe, 2002). Reflecting such natural statistics, a neural population that encodes temporal offsets between actions and sensory feedback would be expected to limit its sensitivity to an applicable range. Therefore we assume that the range of preferred delay in lower level delay neurons is bounded in a range [−*D*, *D*], with *D* as a free parameter.

#### Recalibration of the set-point

The recalibration for TOJs from this circuitry is achieved by incorporating the notion of synaptic scaling, i.e., the “before” and “after” populations have an inherent steady level activity that they attempt to maintain. This assumption is supported by physiological data (Turrigiano, 1999; Turrigiano and Nelson, 2000; Ibata et al., 2008). This is implemented in our model by the following rule:

(2) $$d{\vec{W}}_{q}={\vec{W}}_{q}\cdot \left(f_{\text{ref}}-f_{\text{q}}\right)\cdot \gamma \left(\text{q}=\text{A},\text{B}\right)$$

In the above equation, ${\vec{W}}_{A}$ and ${\vec{W}}_{B}$ denotes the vector of synaptic weights feeding the “after” and “before” pooling model. $d{\vec{W}}_{q}$is the change of weights for each pooling module after one pair of button pressing and flash is observed by the participant *f*_ref_. is the desired steady activity level that both the pooling modules try to maintain, and we assume it is the same for both of the modules. *f*_A_ and *f*_B_ represent the recent activity level of the pooling modules, i.e., the level triggered by the last pair of pressing and flash. γ is the learning rate, a free parameter controlling the speed of weight adjusting. This simple synaptic scaling mechanism makes the pooling modules capable of globally scaling up or down their input synaptic strength if their activities keep deviating from balance. Notice that because the $d{\vec{W}}_{q}$ is proportional to ${\vec{W}}_{q}$, the relative contributions of different delay-tuned neurons’ inputs to each pooling module’s response stay unchanged.

After continuous exposure to an injected delay between motor actions and sensory feedback, the input weights of the “after” module gradually decrease globally, while the weights of the “before” module gradually increase (Figure 3A). This recalibrates the system such that TOJs are biased toward making “before” decision, resulting in a rightward shift of psychometric curve (Figure 3B, with parameters fit in Limited Recalibration with Increased Adapting Delays). We will use this synaptic scaling rule to reproduce the illusory temporal reversal observed by Stetson et al. (2006).

### Procedures of the toj experiments

To assess the performance of the model, we conducted a series of TOJ experiments with human participants. The display was a Viewsonic^®^ G225f CRT monitor with a screen resolution of 1152 × 864 and a refresh rate of 100 Hz. A Matlab^®^ library, Psychtoolbox (Brainard, 1997; Kleiner et al., 2007), was used to present stimuli. Participants sat 50 cm from the screen. They played a game in which they moved the mouse cursor into a circular target on the screen (a “balloon,” spanning 6.9° of visual angle), and popped the balloon with a mouse click (Figure 5A). The balloon pop was indicated by a brief white flash (duration of 10 ms, spanning 13.8° of visual angle). Each new trial started 400 ms after the end of previous trial. The cursor started from the ending location of the last trial and a new target appeared at a random location at least 1/4 of the screen height away from the cursor. In adapting trials, the balloon always popped after a fixed delay, and participants did not need to make any judgment. In test trials, the white flash of the pop happened either before or after the participants clicked the mouse; in these trials, participants reported the order between the click and the flash. Different adapting conditions are described in detail in the Results section, below. A Razer Copperhead^TM^ mouse (response latency ∼5 ms) was used, with all decorating lights removed. Participants wore 33 dB noise reduction earplugs to block the clicking sound generated by the mouse. The implementation of the experiment requires that the onset of the flash can be delivered both before and after the participants click the mouse. This was enabled by predicting the clicking time of the participants.

A time limit of 1 s was imposed for the balloon popping task, so participants would click the mouse as soon as they were sure the cursor was inside the target. Therefore, we could predict the time that participants would click the mouse with reasonable accuracy. To achieve this, the velocity and acceleration of the mouse movement was estimated by a Kalman filter in real time, such that the trajectory of the cursor and the time it would slow-down inside the target area could be estimated on the fly. In addition, the latency from the slowing-down of the mouse to the clicking was estimated from the past trials by a running average. The predicted time of mouse slow-down and the latency before clicking were combined to calculate the predicted clicking time after each refresh of the screen. The popping of the target balloon was presented randomly within a window of −200 to 200 ms relative to the predicted clicking time. Because the latency from mouse slow-down to clicking was also based on the estimated time of mouse slow-down in the past trials, any bias in predicting the time of slow-down was compensated in the estimated latency before clicking. In this way, we were able to test the TOJ with a distribution of click-flash delays that was approximately centered around zero (Figure 5C). The example Matlab codes for these experiments are available at eaglemanlab.net/recalibrationmodel.

The probability of reporting “balloon popped after clicking” given the physical delay between the clicking and the flash was modeled by cumulative Gaussian function (Note that although flashes were delivered based on predicted clicking time, analysis relied on the actual temporal offset between click and flash). The mean and standard deviation of the underlying Gaussian kernel were fitted to the data with maximum likelihood method. The mean of the Gaussian kernel (the delay that participant reported “balloon popped after clicking” at 50% chance) was taken as the point of subjective simultaneity (PSS). The standard deviation of the Gaussian kernel was taken as the just noticeable difference (JND). All the experiments conducted were approved by the Institutional Review Board of Baylor College of Medicine. All the participants gave written consent and received compensation. Model predictions were compared with the data from the psychophysical experiment. The parameters used in the comparisons were derived by fitting the model to the result of Stetson et al., 2006; see Limited Recalibration with Increased Adapting Delays). Therefore the parameters used in the model were independent from all the TOJ experiments conducted in this paper.

## Results

### Model performance

As shown in Figure 3B, the proposed model can successfully predict the shift of the PSS (the offset at which a participant has a 50% chance of judging “flash after press”). When a 100 ms delay is injected between the press and flash in the adapting trials, the observers’ PSSs shift to the right. Here we show that the model can further capture several features of the recalibration in a TOJ task. In addition, the storage of the TOJ recalibration will be demonstrated.

#### Limited recalibration with increased adapting delays

Stetson et al. (2006) demonstrated not only that the subjective simultaneity between action and sensory feedback shifted when participants adapted to a delay between them, but also that the amount of adaptation depended on the length of the adapting delays. For adapting delays of 100, 250, 500, and 1000 ms, the shifts were 44 ± 7, 30 ± 16, 13 ± 16, and −4 ± 16 ms, respectively (values are mean ± SEM). We tested whether our model can also capture this observation of decreasing recalibration with increased adapting delays.

**Figure 4:**
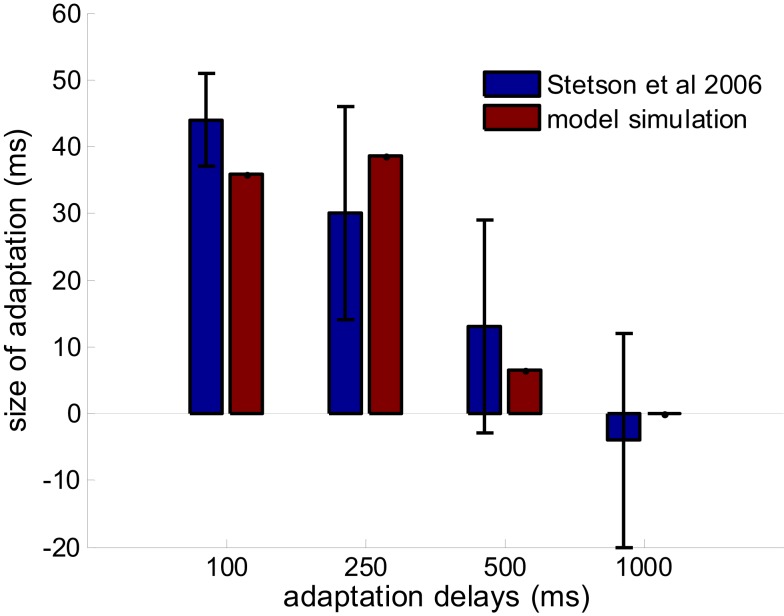
**Prediction of the model on the limited adaptation size for large adaptation delay (red bars)**. Results from Stetson et al., 2006 are reproduced in blue bars with permission (*n* = 25, 5, 4, 4 for the adapting delays of 100, 250, 500, and 1000 ms). The delay-tuned neurons have a limit on the offsets that they can encode, which gives rise to this limited capacity of recalibration.

In the simulation of TOJ, the distribution of delays between button pressing and flash mimics the experiments in Stetson et al. (2006). Namely, after 2–6 trials of a constant adapting delay, a testing trial with random delay from −200 to 200 ms is fed to the model. In the control condition, the constant delay in adapting trials is 0 ms. In the adaptation condition, the constant delays are 100, 250, 500, and 1000 ms; each adaption delay is evaluated in a separate simulations. The delay between the button press and the flash in each trial is the input to all of the lower level “delay neurons.” The modeled network then makes a judgment for each trial and recalibrates the synaptic weights to the pooling module according to the equations articulated above. A psychometric curve is fit to the judgments that the pooling modules made for the test trials. The shift of PSS from the control condition to the adaptation condition is compared against the shift reported in Stetson et al. (Figure 4).

As mentioned, there are four free parameters in the model: the learning rate for synaptic scaling (γ), the range of optimal delays for the lower level “delay-tuned neurons” ([−*D*, *D*]), the width of their tuning curves (σ), and the standard deviation of the cumulative Gaussian function describing the weight distribution (λ). The distance between the optimal delays of two adjacent simulated neurons is set to 20 ms. The maximum firing rate for the delay-tuned neurons is set to 100 Hz. The model is simulated with parameter sets on grids spanning a large range in the parameter space; each parameter set on the grid is simulated 40 times. The parameter that generates the least root mean square error on predicting the size of adaptation by different adapting delays reported in Stetson et al. (2006) is chosen as the optimal parameter set.

The optimal parameters are found as following: 6 × 10^−4^ for learning rate (γ), 40 ms for tuning width (σ), 30 ms for (λ), and [−440, 440] ms as the range of optimal offsets ([−*D*, *D*]). This range also falls in to the time window for multisensory integration predicted by other theoretical work (Colonius and Diederich, 2010), and coincidentally, very close to the maximum range of intervals from motor-sensory events that DLPFC and CN neurons have peak response to (Jin et al., 2009). Results comparing the data from Stetson et al., 2006 and the simulation are shown in Figure 4. The size of adaptation (that is, the shift of the PSS between conditions) is obtained by averaging 400 repeated simulations with the optimal parameter set. The relation between the average shifts and adapting delays can be well captured by the model. In fact, the model is not very sensitive to the parameter selection. By varying all the parameters by ratios from 0.67 to 1.5, the predicted shifts of PSS for the four adapting delays are 36 ± 15, 41 ± 21, 19 ± 19 and 0 ± 1 ms, respectively (mean ± STD). The main variation of the prediction is contributed by the variation of the tuning width of lower level neurons.

The model predicts a small increase of JND (defined as the standard deviation of the Gaussian kernel underlying the psychometric function), from 50 to 59 ms after adapting to 100 ms delay. Stetson et al. (2006) found no significant change in the slope of the psychometric curves after adaptation. The average JND across baseline and adaptation conditions in Stetson et al. (2006)’s data was 46 ± 7 ms (mean ± SEM, unpublished). Note that although the parameters are only optimized to fit the shifts of PSS, without regard of the JND, the prediction of JND also falls in the range of the psychometric data.

#### Dependence of the size of recalibration on adapting/test trial ratio

According to our recalibration model, the pooling modules adjust their input weights based on their activity after each input. Therefore, the test stimuli with variable delays between action and sensation also contribute to the set-points of the TOJ system in addition to the influence of the constant injected delay in the adapting trials. One natural prediction of the model is that the size of recalibration, represented by the shift of the PSS, increases as participants are exposed to more adapting trials before a test trial.

**Figure 5:**
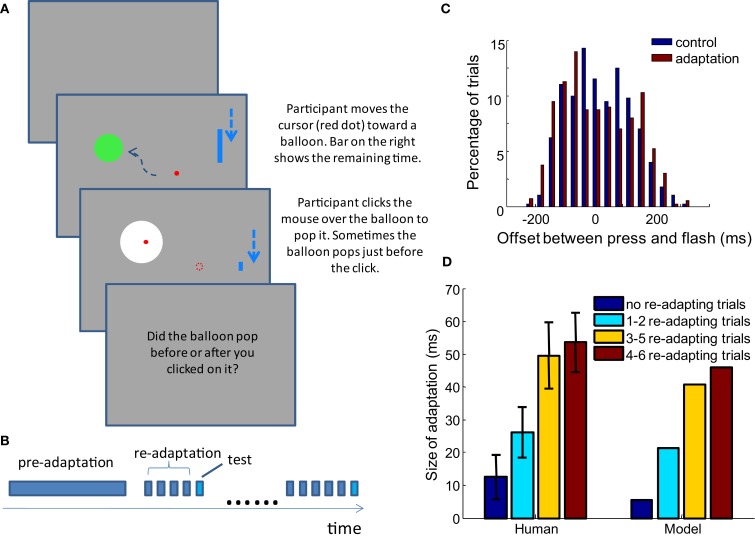
**The size of adaptation increases as participants are exposed to more re-adapting trials before each test trial, as predicted by the model**. **(A)** Task of the experiment: participants control the red dot with the mouse, and hit the static green disc representing a balloon. A shrinking blue bar on the right indicates the time left for popping the balloon. In adapting trials, if the participant presses before the time bar shrinks to bottom, the balloon pops (color change to white) with constant delay after the press. In test trials it pops before or after the press is made. Participants only need to judge the order of click and flash for test trials. In the real experiment, the dashed arrows are not seen. **(B)** Structure of an experiment block: 50 pre-adaptation trials lead to testing phase, in which each test trial is preceded by a random number of re-adapting trials (no such re-adapting trial in 0 re-adapting trial condition). **(C)** The distribution of the temporal offset for test trials is well balanced in both the control and adaptation blocks. The figure shows the distribution in the 3–5 adapting trial condition across all participants. **(D)** Using the parameter set obtained in Limited recalibration with increased adapting delays, the model successfully captures the average result of behavioral data. Error bars = SEM. *n* = 31, 16, 18, 15.

To test this hypothesis, we conducted an experiment in which the amount of adaptation before each test trial is controlled. The TOJ task in form of the “balloon popping game” described in Materials and Methods is used. An experiment starts with 50 adapting trials that do not require judgment, followed by the testing phase, in which none or several adapting trials precede a test trial (Figure 5B). For clarity, the initial adapting trials at the beginning of the experiment are termed pre-adapting trials, and the adapting trials before each test trial are termed re-adapting trials (other groups sometimes call these “top-up” trials). Four groups of participants were tested with different conditions distinguished by the number of re-adapting trials preceding a testing trial. In each condition, 0, 1–2, 3–5, or 4–6 re-adapting trials were presented before each testing trial.

In each condition, there is a control block and an adaptation block, each of which includes 60 test trials (in the 0 and 1–2 re-adapting trial conditions, 100 trials are tested but only the first 60 trials are analyzed, consistent with other re-adapting number conditions). The structures of the control and adaptation blocks are the same, as described above and shown in Figure 5B. The difference is that in the adaptation block, a 100 ms delay is injected between the movement of the mouse and the movement of the cursor, as well as between the mouse-clicks and flashes in all the adapting trials. In the control block, the delays (both mouse cursor and click-flash) are only 10 ms, corresponding to the interval between two frames on the monitor. 33, 16, 25, and 16 healthy participants took part in the experiments of the four re-adapting conditions respectively. PSS and JND are calculated in each condition for every participant. If the obtained PSS was outside the main range of the offsets tested (−200 to 200 ms) or the JND was >150 ms (indicating poor judgment of temporal order), the participant’s data were excluded from further analysis. Data from 31, 16, 18, and 15 participants of the four re-adapting conditions were retained with this criterion. The difference between the PSS in the adaptation block and the control block in each re-adapting number condition is shown in Figure 5D, together with the prediction of the model.

The model prediction is based on the optimal parameter fitted for the result of Stetson et al. (2006), averaged over 400 repeated simulations. Therefore there is no parameter adjustment for the current experimental data. As predicted by our model, the shift of PSS increases with the ratio between the number of re-adapting trials and the number of test trials. The shifts of PSS in the psychophysical data are not significantly different from the shifts predicted by the model in any of the four conditions (*t*-test, *p* = 0.31, 0.54, 0.40, and 0.41, respectively).

#### Storage of recalibration of TOJ

The MAE is generated when an observer adapts to persistent visual motion. It gradually diminishes as observers watch a static image or a random kinematogram for a period of time after adaptation. However, if observers keep their eyes closed after adapting to visual motion, the aftereffect still exists once they open their eyes – an effect known as *storage* (Mather et al., 1998).

If the same neural mechanism underlies both MAE and recalibration of TOJ, then we expect storage also occurs for the TOJ recalibration. And indeed, storage is predicted by Eq. 2, because the synaptic scaling only happens after each pair of motor-sensory events is observed, and the scaled synaptic weights will remain unchanged until the next observation. Therefore, according to the model, if participants in the balloon popping game were to keep their hands still and look at blank visual background for several seconds after adaptation, the recalibration should remain stored and observable when they are next tested.

**Figure 6:**
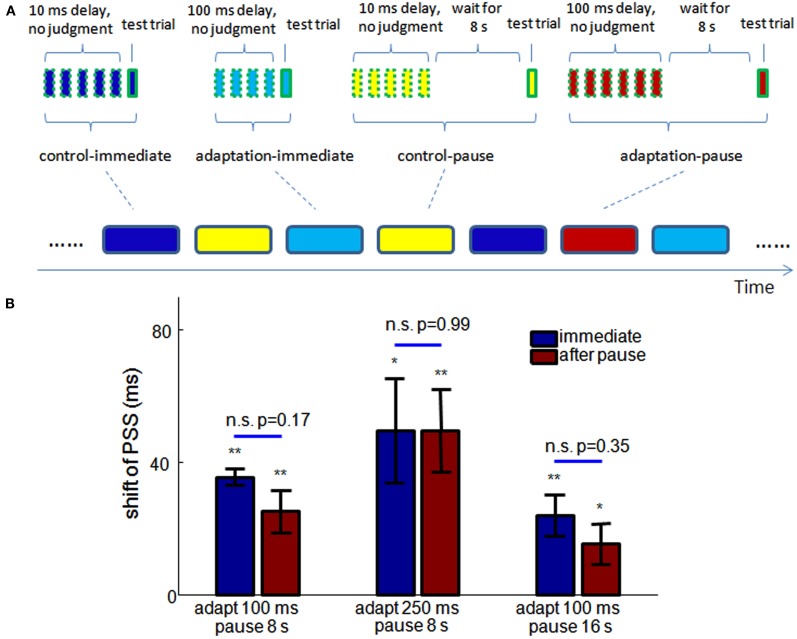
**Storage of TOJ recalibration**. **(A)** Experimental design. Meta-trials of four different conditions (indicated by the round-corner rectangles at the bottom) are randomly interleaved in the experiment. Each meta-trial consists of 4–6 adapting trials (squares of dashed outline) and one test trial, with additional 8 s wait time in the control-pause and adaptation-pause conditions (the pause is 16 s in the third experiment). **(B)** The shifts of PSS from control to adaptation conditions in the three experiments (*n* = 10, 10, 9). No significant difference of the shifts of PSS is observed with and without a pause before a test trial, in any of the experiments. Blue: test trial immediately follows adapting trials. Red: a pause is inserted between adapting trials and the test trials. *significantly different from 0, *p* < 0.05, **p < 0.01. Errorbar: SEM.

To test this prediction in humans, we conducted new experiments with the balloon popping game. As shown in Figure 6A, the experiment consisted of randomly interleaved “meta-trials” of four conditions: control-immediate, adaptation-immediate, control-pause, and adaptation-pause. Each meta-trial had 4–6 adapting trials before one test trial. In the adapting trials of the control-immediate and control-pause meta-trials, the delay between mouse-and-cursor, and between click-and-flash were both 10 ms. In the adapting trials of the adaptation-immediate and adaptation-pause meta-trials, the injected delay was 100 ms. The flash occurred either before or after the clicking in the test trials, the same as in Dependence of the Size of Recalibration on Adapting/Test Trial Ratio. In the control-immediate and adaptation-immediate meta-trials, the test trial followed the adapting trials immediately, while in the control-pause and adaptation-pause meta-trials, the test trial started after a pause of 8 s, during which time participants were instructed to not move their head or hands, and to look at the blank computer screen. Two sessions of experiments were tested on two different days for each participant. In each session, 48 meta-trials were tested for each condition.

Psychometric functions were estimated for each condition for each participant. The shift of PSS from control-immediate to adaptation-immediate conditions and from control-pause to adaptation-pause conditions are shown by the leftmost blue and red bars. The shifts of PSS are not significantly different (*p* = 0.16) with and without a pause before the test.

In addition, we conducted two further experiments to test the ubiquity of storage across adapting delay and the lifetime of storage. In the second experiment, the adapting delay was changed from 100 ms to 250 ms for both the adaptation-immediate and adaptation-pause conditions. In the third experiment, the adapting delay was still 100 ms but the pause in the control-pause and adaptation-pause conditions were elongated to 16 s. The trial number for each meta-trial condition was reduced to 42 during each session (two sessions for each experiment) due to increased experiment time. 10, 11, and 11 participants were tested for the three experiments in Figure 6B, respectively. With the same criterion as in Dependence of the Size of Recalibration on Adapting/Test Trial Ratio, 1 and 2 participants were excluded in the second and third experiments from further analysis.

The shifts of PSS in these two experiments are shown in the middle and right groups of bars of Figure 6B. Again, there is no significant difference in the shifts of PSS between immediate and pause conditions in either of the experiments. A 2-way unbalanced ANOVA was used to test the effect of the adapting delay and pause period across the three storage experiments. There is a significant effect of the adapting delay on the shift of PSS (*p* = 0.016), but no significant effect of the pause period (*p* = 0.49).

This result demonstrates that 4–6 exposures to consistent delay between motor action and sensory feedback are sufficient to induce recalibration of subjective temporal order. Otherwise, if the recalibration were slow, the randomized sequence of meta-trials should eliminate the difference between control and adaptation conditions. Further, although the set-point of a TOJ can change rapidly, it is not due solely to the decay of neural fatigue over time. The 8 s pause in two of the pause conditions is longer than the time taken by 4–6 adapting trials (around 5–8 s), but it does not remove the effect of recalibration. In other words, the recalibration is stored only if no further evidence of a temporal relation between the action and sensation is provided to the system. Figure 6B further demonstrates that the storage of recalibration holds for longer adapting delays and only shows a slight (and insignificant) decrease after a 16 s pause. No significant difference is found between the JNDs of any two conditions in each experiment (paired *t*-test).

#### Relation of the model to motion direction judgments

As shown by our findings above, the recalibration of the TOJ appears to be a temporal analog of the MAE. Our opponent processing model for a TOJ is inspired by the framework set up in Shadlen et al. (1996) for the motion direction judgment. Here we show that by combining information pooling, opponent processing, and synaptic scaling on the motion direction judgment, we can create a new model, similar to what we proposed for TOJ, which can also explain the MAE. We used a traditional stimulus for studying motion perception: the random dot kinematogram. As described in previous work (Shadlen et al., 1996), dots move randomly in different directions on a computer screen, with a variable proportion of the dots coherently moving leftward or rightward. Participants were asked to judge whether more dots moved toward the left or toward the right. Psychometric curves were fit to the frequency that participants reported the dots moving in one direction as a function of the proportion of the coherently moving dots. The MAE is observed when each brief test stimulus is preceded by a prolonged stimulus with all dots moving in one direction. Participants have higher probability of judging the brief test stimulus as moving in the opposite direction of the prolonged adapting stimulus.

We used the results found in Shadlen et al. (1996) to model the opponent process in motion direction judgments. In this motion version of our model, low-level neurons are tuned to different directions of visual motion (similar to Figure 2A, but here the neurons are tuned for motion direction instead of temporal offsets between action and sensation). The activity of these low-level neurons are in turn pooled by two modules that compete with each other (for example, for “leftward” and “rightward”). The pooling module that has higher activity indicates the direction of the global visual motion.

The activity of the *i*^th^ direction tuned neuron is modeled as the sum of the contribution of each moving dots in its receptive field:

$$x_{i}=F\frac{1}{m}\overset{m}{\underset{k=1}{\sum }}z_{i}\left(\beta_{k}\right)+\varepsilon$$

*F* is the gain factor, corresponding to the maximal firing rate. *m* is the number of dots β*_k_* is the motion direction of the *k*^th^ dot. ε represents additive noise approximating a Poisson noise (Fano factor is 1).

$z_{i}\left(\beta_{k}\right)$ is the contribution of the moving dot *d_k_* to the activity of the *i*^th^ neuron. As direction is a parameter defined on circular range, an appropriate way to describe a tuning curve mathematically is by a von Mises distribution (Swindale, 1998; Ringach et al., 2003):

$$z_{i}\left(\beta_{k}\right)=\exp\left\{\kappa \left[\cos\left(\theta_{i}-\beta_{k}\right)-1\right]\right\}$$

θ*_i_* is the *i*^th^ neuron’s preferred direction. β*_k_* is the moving direction of dot d*_k_*. κ is a factor characterizing the concentration of the tuning curve. Larger κ indicates narrower tuning curve. The population response of the motion tuning neurons is denoted as $\vec{X}=\left[x_{1},\;x_{2},\;x_{3},\;...,\;x_{n}\right]$.

Similarly, the firing outputs of the motion tuning neurons are pooled by two modules. The activities of the pooling modules are denoted as:

$$f_{q}=\vec{X}\cdot {\vec{W}}_{q}\left(q=\text{L,}\;\text{R}\right).$$

${\vec{W}}_{L}$ denotes the input weights of the “left” pooling module that receives stronger input from the low-level neurons of which the preferred motion direction has a leftward component. ${\vec{W}}_{R}$ denotes the input weights of the “right” pooling module that receives stronger input from the rest of the low-level neurons.

$${\vec{W}}_{q}=\left[w_{q1},w_{q2},w_{q3},....,w_{qn}\right],\;\left(q=\text{L,}\;\text{R}\right)$$

We use the cosine function to model such uneven weight distributions:

$$\begin{array}{llll} & w_{Li}=\cos\left(\theta_{i}-180^{\circ }\right)+1 & & \\ & w_{Ri}=\cos\left(\theta_{i}\right)+1 & & \end{array}$$

w_Li_ and w_Ri_ are the input weights of the *i*^th^ motion direction sensitive neuron to the “left” and “right” pooling modules, respectively. 0° represents rightward and 180°corresponds to leftward direction.

Input weights are updated with the same synaptic scaling rule as in the TOJ model:

$$d{\vec{W}}_{q}={\vec{W}}_{q}\cdot \left(f_{\text{ref}}-f_{q}\right)\cdot \gamma$$

where *q* = L, R.

In the temporal case, the opponent processing occurs after a participant clicks a mouse and sees a flash, while in motion case the opponent processing occurs while observing the stimulus. Thus, to directly compare the two cases, we modeled a prolonged adapting stimulus of moving dots as several consecutive short stimuli with the same durations as the test stimuli. In this way, each short period during the adapting motion can be considered equivalent to a single trial in the temporal case.

**Figure 7:**
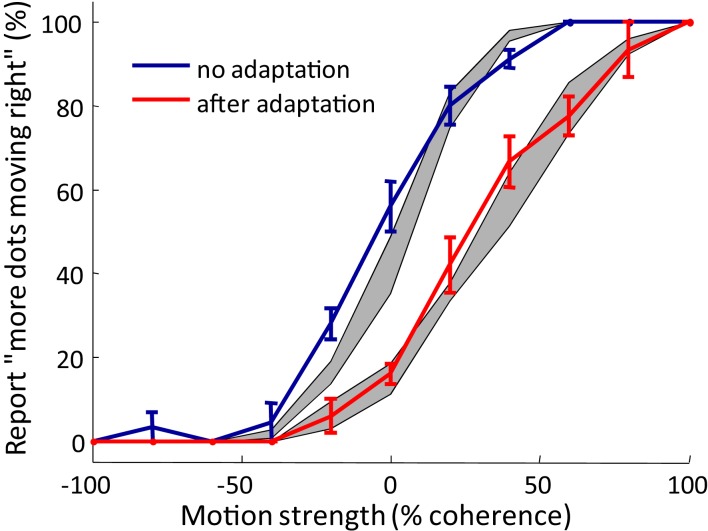
**Typical psychometric curves showing motion aftereffect, as tested with random dot kinematograms**. Solid lines: psychometric curves averaged over participants (*n* = 5). Error bar = SEM Red curve: adaptation condition in which participants view 15 s of dots moving rightward before each test stimulus of varying coherence that lasts 0.5 s. Blue curve: control condition in which there is no adapting stimulus preceding the test stimulus. Grey areas: simulation result from the model in Relation of the model to motion direction judgments (mean ± SEM). Repeat = 5.

We tested the performance of the model by comparing the predicted MAE with the behavior of human participants. Participants judged the global motion of the kinematogram stimulus of different coherence levels, each lasting 0.5 s. As expected, if a 15 s adapting stimulus of dots coherently moving rightward was presented before each test stimulus, the psychometric curve shifted toward more “leftward” judgments. Our model prediction of the psychometric curves with and without adaptation matches the data from human observers (Figure 7).

## Discussion

The model presented in this paper, based on neural motifs of information pooling, opponent processing, and synaptic scaling, successfully explains the recalibration in the temporal order and motion direction judgment tasks. The performance of the model in TOJs is further validated by predicting the relation between the recalibration size and re-adapting/test ratio based on model parameters fit independently to the results of previous literature (Stetson et al., 2006). We found that the recalibration is measurable after 4–6 exposures to a constant motor-sensory delay, but this recalibration does not diminish with the same amount of time without further exposure.

One account of the recalibration after adaptation to audiovisual asynchrony suggests that either the audio or visual event is delayed or sped up after adaptation (Di Luca et al., 2009; Navarra et al., 2009). This account relies on an assumption of an internal time line on which events from different modalities have to be time-stamped in order to be compared. Here we provide an alternative mechanism that does not rely on such an internal time line. Instead, the model presented here relies on three broadly observed neural mechanisms: neural pooling, opponent processing, and synaptic scaling. Neural populations that encode for temporal disparity between motor efference copy and sensory modality is assumed in the model. In fact, neurons sensitive to delays between events from different modalities have been found in cats (Meredith et al., 1987). It is possible that similar delay sensitive neurons also exist to detect temporal disparity between neural activity in one sensory pathway and the efference copy of a motor command.

Although in the current paper, the experimental data by which the model is fit and the current psychophysics experiments are focused on motor-sensory TOJ, our model is not limited to this domain. The same principle can be used to explain the similar recalibration found in multisensory asynchrony adaptation such as in Vroomen et al. (2004). Slight modification of the decision rule can make the model suitable to simultaneity judgment tasks (Fujisaki et al., 2004) where a monitoring module makes a decision that the audio and visual inputs are simultaneous if the difference between the responses of the two pooling populations is smaller than a threshold. As long as the same synaptic scaling rule is employed by the two pooling modules, the set-point that the responses of the two pooling modules balance would shift by constantly adapting to stimulus asynchrony.

The finding of the storage of recalibration is consistent with our hypothesis that the same neural mechanisms may underlie perceptual judgments in both space and time. Speculatively, this suggests the possibility of a genetic program capable of unpacking a module that can effectively deal with either time or space depending only upon the inputs. To put it another way, such a mechanism may represent a meta-modal operator: a circuit that performs the same basic operations irrespective of its input (Pascual-Leone and Hamilton, 2001). In this case, we are hypothesizing that the basic operation of pooling and opponent processing can perform useful operations underlying perceptual judgments in both space and time.

Storage in the recalibration of audiovisual stimuli has also been shown recently (Machulla et al., 2012). The results of our experiments provide the first evidence that recalibration of motor-sensory TOJs can be stored. Although the experiment paradigms are not identical between Machulla et al. (2012) and ours, together they suggest that the storage of TOJ recalibration is ubiquitous across tasks and modality. However, it is noteworthy that the recalibration of temporal order perception is larger and more rapid in motor-sensory tasks than it is in cross-sensory tasks (Stetson et al., 2006).

Other studies have recently specified how neural mechanisms can underlie TOJs. In a model by Roach et al. (2011), a TOJ is achieved by comparing the maximum posterior estimate of delay with a standard neural representation of simultaneity (Roach et al., 2011). Further, they suggest that the aftereffect of adapting to audiovisual asynchrony stems from the decrease of response gain of the neurons that are most sensitive to the adapted delay. It is worth pointing out the differences with our model: in the framework we have presented, a TOJ results from competition between two pooling modules, and the aftereffect arises from the global change of synaptic strengths of the pooling modules. Both their model and ours point to a possible organization of the brain: the same type of circuit may be used in different brain regions, and the model functions depend on the input to the circuits. For example, here we show that the same mechanism could underlie both the recalibration of TOJ task and the MAE. Similarly, the model by Roach et al. also applies to the tilt aftereffect. Both of the models seem able to explain the recalibration from the existing data. The data of the current experiments are not yet sufficient to reject either of the models. In order for the model of Roach et al. to catch the fact that recalibration is measurable with interleaved meta-trials, the neural gain reduction due to repeated exposure to the same delay needs to be paired with a recovery mechanism. On the other hand, in order to explain storage of recalibration, the recovery needs to mainly depend on the further sensory evidence following gain reduction, instead of merely on the time elapsed. The synaptic scaling proposed here appears similar to a bi-directional neural gain control mechanism on the level of the pooling neurons. As a single principle, it saves the necessity to specify the dynamics of recovery if uni-directional neural gain reduction is assumed. Phenomenally, the behavior of the higher level pooling neurons in our model seems like simple, broadly tuned interval sensing neurons. We did not attempt to simplify the model by removing the lower level neurons, because the existing findings of the interval tuning neurons (Meredith et al., 1987; Jin et al., 2009) mainly show the narrowly tuned neurons as the lower level ones in our model. Our model provides just one type of computation that can be performed based on this type of tuning neurons and does not preclude other functions that these tuning neurons can support.

It is noteworthy that even in the spatial domain, the MAE may have multiple components and can involve neural changes in different levels (Mather et al., 2008). It is also possible that multiple mechanisms are involved in the cross-sensory and motor-sensory recalibration of simultaneity, including change of processing latency, neural gain, or pooling synaptic strength. Much of our model is motivated by the findings in electrophysiological recordings in motion-selective neurons and the models for motion adaptation (e.g., Shadlen et al., 1996). There are several theories on the functional role of adaptation in the spatial domain (Mather, 1980; Wainwright, 1999; Kohn, 2007). The idea of our model is similar to that of error correction (Anstis, 1975): if sensory events are often perceived as coming after an action, there is likely to be some error of temporal order perception – and so the system needs to adjust the pooling module in order to correct this error. The research on interval tuning neurons is still very limited compared to the study on neurons encoding for spatial features. The similar properties in psychophysics of space and time perception raise the possibility that common mechanisms may be shared between the two domains. We suggest that many of the research paradigms used in spatial perception may help us to better understand the mechanisms of time perception. Further theoretical and electrophysiological explorations are crucial for this field.

## Conflict of Interest Statement

The authors declare that the research was conducted in the absence of any commercial or financial relationships that could be construed as a potential conflict of interest.
